# Exploration of factors influencing pulmonary injury in patients with anti-neutrophil cytoplasmic antibody-associated vasculitis and construction of a predictive model

**DOI:** 10.3389/fmed.2025.1724495

**Published:** 2026-01-02

**Authors:** Linlin Zheng, Chongyang Liu

**Affiliations:** Department of Rheumatology and Immunology, The Third Affiliated Hospital of Chongqing Medical University, Chongqing, China

**Keywords:** ANCA-associated vasculitis, pulmonary involvement, predictive model, risk factors, LASSO regression, hemoglobin, intermittent symptoms

## Abstract

**Background:**

A bad prognosis results from pulmonary involvement of anti-neutrophil cytoplasmic antibody-associated vasculitis (AAV). A method for early detection of AAV patients at risk of developing pulmonary involvement is still required, despite the existence of predictive models for death. The purpose of this study was to determine risk factors and develop a predictive model for pulmonary involvement in AAV.

**Methods:**

Seventy-one treatment-naïve AAV patients (38 with pulmonary involvement) participated in a retrospective cross-sectional investigation. To choose predictors and create a nomogram, multivariate logistic regression and the least absolute shrinkage and selection operator (LASSO) were employed. Receiver operating characteristic (ROC) curves, calibration plots, and decision curve analysis (DCA) with a 7:3 training-to-validation split were used to assess the model.

**Results:**

Four independent predictors were identified: intermittent symptom pattern (OR = 8.52, 95% CI: 2.17–33.46), limb numbness/paresthesia (OR = 12.21, 95% CI: 1.98–75.50), older age at onset (OR = 1.05 per year, 95% CI: 1.01–1.10), and lower hemoglobin level (OR = 0.97 per g/L, 95% CI: 0.95–0.99). A nomogram incorporating these factors demonstrated good discrimination, with an area under the curve (AUC) of 0.88 (95% CI: 0.77–0.98) in the training set and 0.83 (95% CI: 0.64–1.00) in the validation set. Calibration and decision curve analysis confirmed the model’s clinical utility.

**Conclusion:**

Using standard clinical markers, we created and internally verified a pragmatic prediction model for pulmonary involvement in AAV. Personalized monitoring and early risk categorization may benefit from this approach. To verify its generalizability, external validation in prospective, multi-center cohorts is advised.

## Introduction

Anti-neutrophil cytoplasmic antibody-associated vasculitis (AAV) is a systemic autoimmune disease that affects multiple organ systems. Its primary pathogenic mechanism involves the excessive activation of neutrophils by ANCA, leading to abnormal interactions between activated neutrophils and endothelial cells, ultimately causing vasculitic lesions. AAV encompasses three main subtypes: microscopic polyangiitis, granulomatosis with polyangiitis, and eosinophilic granulomatosis with polyangiitis (1). The hallmark features of AAV include systemic inflammatory reactions and fibrinoid necrosis in the walls of small blood vessels, accompanied by positive serum ANCA. However, unlike other vasculitides, immune complex deposition is typically absent in the affected vascular walls (2).

Over the past 30 years, studies have demonstrated a significant increase in the incidence of AAV (2). In 2008, the annual overall incidence of ANCA-associated vasculitis in Europe and North America was approximately 20 cases per million (3). By 2015, the overall incidence had risen to 420/125,000 (2). In China, a sample survey of hospitalized patients reported an incidence rate of approximately 0.025% for AAV (4). It has been reported that untreated granulomatosis with polyangiitis (GPA) has a one-year mortality rate as high as 80%, with an average survival time of about 5 months (5).

AAV predominantly affects middle-aged and elderly individuals and typically has an insidious onset. The disease often alternates between periods of remission and exacerbation, lacks specific clinical manifestations, and primarily involves the lungs, kidneys, and nervous system. In these patients, 85–90% of those with GPA will develop pulmonary involvement, which manifests as pulmonary hemorrhage or interstitial pulmonary fibrosis. In contrast, the incidence of pulmonary damage in microscopic polyangiitis (MPA) is slightly lower, ranging from 25 to 55% (6). Moreover, in patients with MPA, approximately 30–50% of those diagnosed develop pulmonary involvement within 1 year. The corresponding proportions are up to 60% in EGPA (95% including asthma) and 67 to 85% in GPA (7). Pulmonary injury can exhibit significant variability and progress rapidly, ranging from asymptomatic cases to severe pulmonary hemorrhage, dyspnea, and respiratory distress, potentially resulting in patient mortality. In patients with severe pulmonary injury, the 3-month mortality rate is 23%, while the 12-month mortality rate is 26% (8).

While the variability in pulmonary involvement and its associated high mortality rates highlight the clinical significance of early intervention, the lack of consensus on the risk factors contributing to this condition presents a major challenge. Understanding these factors is crucial for the development of predictive tools that could facilitate timely diagnosis and targeted treatment, ultimately improving patient outcomes. Therefore, we designed a retrospective study to explore the correlations between various clinical and pathological indicators and the occurrence of pulmonary involvement in AAV patients, and to establish a predictive model that can aid clinicians in making timely and informed medical decisions.

## Materials and methods

### Study design

This retrospective cross-sectional study was conducted using clinical data from patients diagnosed with AAV who were hospitalized at the Third Affiliated Hospital of Chongqing Medical University between August 2018 and October 2024. The study protocol was approved by the hospital’s Ethics Committee (Approval No.: 2025-Kelun-2). Informed consent was waived due to the retrospective nature of the study and the use of fully anonymized clinical data. Our final cohort included 71 eligible treatment-naïve AAV patients (38 with pulmonary involvement). Model development and reporting followed the TRIPOD guidelines (9).

### Participant selection

A total of 71 AAV patients were included, comprising 38 with pulmonary involvement and 33 without. Diagnosis of AAV was based on the 2012 Chapel Hill Consensus Conference criteria (1, 10, 11), supported by serological ANCA positivity and/or histopathological evidence of necrotizing vasculitis. Both ANCA-positive and ANCA-negative patients with histologically confirmed renal vasculitis were eligible.

*Inclusion criteria*: (1) Age ≥ 18 years; (2) New diagnosis or diagnosis within 1 year of AAV, with no prior treatment with glucocorticoids, immunosuppressants, or biologics; (3) Complete clinical records available.

*Exclusion criteria*: (1) Age < 18 years; (2) Secondary vasculitis due to drugs, malignancies, or infections (e.g., tuberculosis); (3) Coexisting autoimmune diseases (e.g., systemic lupus erythematosus, rheumatoid arthritis, anti-GBM nephritis); (4) Pre-existing pulmonary diseases (e.g., tumors) or history of pulmonary injury due to infection, drugs, or environmental factors prior to AAV onset.

### Diagnostic criteria for pulmonary involvement

Pulmonary involvement was defined according to established composite criteria (7, 12, 13): (a) Clinical symptoms (e.g., hemoptysis, cough, dyspnea); (b) Imaging findings consistent with alveolar hemorrhage, interstitial changes, nodules, cavitation, or tracheobronchial inflammation; (c) Pulmonary function impairment, defined as forced expiratory volume in 1 s (FEV₁) < lower limit of normal (LLN) or < 70% predicted, FEV₁/forced vital capacity (FVC) < LLN, or diffusing capacity of the lung for carbon monoxide (DLCO) < 80% predicted or below LLN (14). All imaging studies were reviewed by a multidisciplinary team including radiologists, pulmonologists, and rheumatologists.

### Data collection

Data were systematically extracted from electronic medical records using a standardized data collection form. All variables were predefined and categorized as follows:

*General information*: Sex (recorded as male or female), age at onset (in years), smoking history (categorized as never smoker or ever smoker, where ever smoker was defined as having smoked at least 100 cigarettes in their lifetime), hypertension (no/yes), diabetes (no/yes), renal function markers, and urinary protein levels (classified as negative, 1+, 2+, or 3+).

*Biochemical parameters were assessed using standardized laboratory procedures*: Hemoglobin (HGB, reported in g/L) was measured by colorimetry using automated hematology analyzers (Sysmex XN-series) as an indicator of anemia. White blood cell count (WBC) and platelet count (PLT) were quantified via flow cytometry and impedance-based methods, respectively, on the same platform to evaluate inflammatory status and coagulation function; High-sensitivity C-reactive protein (hs-CRP) was analyzed by immunoturbidimetry (Roche Cobas c501) as a marker of systemic inflammation; Alanine aminotransferase (ALT), and aspartate aminotransferase (AST) were determined enzymatically (Roche Cobas c501) to assess liver function; Serum creatinine was measured using an enzymatic assay for renal function evaluation; Complement components C3 and C4 were detected via immunoturbidimetry (Siemens BN II) to monitor complement activity; Rheumatoid factor (RF) was quantified by immunonephelometry (Siemens BN II) as an indicator of autoimmune activity; Neutrophil percentage (%) was derived from automated differential counts of peripheral blood smears to estimate acute inflammation.

*Clinicopathological factors*: The following clinicopathological factors were documented. (1) Lung infection within 1 year prior to AAV diagnosis was recorded as present or absent based on corresponding ICD-10 diagnostic codes (15). (2) Symptom frequency during treatment was classified as either intermittent or persistent. Intermittent was defined by symptoms occurring in discrete episodes with intervening remission periods of at least 2 weeks, whereas persistent was defined by symptoms that were continuously present. This assessment covered the six-month period preceding diagnosis. (3) Limb numbness or paresthesia was recorded as present or absent based on documentation in the medical records.

All data extraction was conducted independently by two researchers. Any discrepancies were resolved through consensus or by consultation with a third reviewer. The primary outcome was the presence of pulmonary involvement at the time of AAV diagnosis, defined according to composite criteria incorporating clinical symptoms, imaging results, and pulmonary function tests, as detailed in Section 2.3 (Diagnostic criteria for pulmonary involvement).

### Statistical analysis

Categorical variables are presented as frequencies and percentages, continuous variables as mean ± standard deviation (SD) or median with interquartile range (IQR), based on distribution. Group comparisons used chi-square or Fisher’s exact test for categorical variables, and Student’s t-test or Mann–Whitney U test for continuous variables. A two-tailed *p* < 0.05 was considered statistically significant.

Variable selection was performed using Least Absolute Shrinkage and Selection Operator (LASSO) regression with 10-fold cross-validation (R version 4.2.3, glmnet package 4.1.8). Univariate logistic regression was used to further investigate the predictors found by LASSO. In order to evaluate their independent associations with pulmonary involvement, variables with *p* < 0.05 in univariate analysis were added to a multivariate logistic regression model (SPSS 25.0). The results were presented as odds ratios (ORs) with 95% confidence intervals (CIs).

Restricted cubic spline (RCS) analysis with 3 knots was used to examine the non-linear relationship between hemoglobin (continuous) and pulmonary involvement, adjusted for age (R package rms). A nomogram was constructed based on the final multivariate model. Model performance was evaluated using receiver operating characteristic (ROC) curves, calibration plots, and decision curve analysis (DCA). The dataset was randomly split into training (70%) and validation (30%) sets. To further mitigate overfitting and obtain robust performance estimates, internal validation was performed using 500 bootstrap samples on the training set.

## Results

### General characteristics and univariate analysis of AAV patients with or without pulmonary involvement

A total of 71 patients diagnosed with AAV were included in the analysis, of whom 38 had developed pulmonary involvement and 33 had not (see Table 1). The age of onset was significantly higher in the pulmonary involvement group (66.61 ± 8.95 years) than in the non-pulmonary involvement group (55.09 ± 19.69 years; *t* = −3.09, *p* = 0.003). Hemoglobin levels also differed significantly between the groups, with the pulmonary involvement group displaying lower levels (89.97 ± 22.12 g/L) compared to the non-pulmonary involvement group (105.61 ± 27.19 g/L, *t* = 2.67, *p* = 0.009). In contrast, no significant differences were identified for other variables, including complement C4 levels, quantitative immunoglobulin (IgG), and erythrocyte sedimentation rate (ESR) (all *p* > 0.05).

**Table 1:** General characteristics and clinicopathological factors of patients with and without pulmonary involvement in AAV.

Variables	Total (*n* = 71)	No (*n* = 33)	Yes (*n* = 38)	Statistic	*p*
Age of onset (years), Mean ± SD	61.25 ± 15.91	55.09 ± 19.69	66.61 ± 8.95	*t* = −3.09	0.003
Complement C4 (mg/dL), Mean ± SD	21.84 ± 9.93	22.36 ± 7.76	21.38 ± 11.60	*t* = 0.37	0.715
Quantitative Immunoglobulin (IgG, g/L), Mean ± SD	15.14 ± 6.25	14.37 ± 6.85	15.79 ± 5.74	*t* = −0.83	0.408
Red Blood Cell count (RBC, 10^12/L), Mean ± SD	3.51 ± 0.78	3.64 ± 0.87	3.41 ± 0.70	*t* = 1.23	0.224
Hemoglobin (HGB, g/L), Mean ± SD	97.24 ± 25.66	105.61 ± 27.19	89.97 ± 22.12	*t* = 2.67	0.009
Neutrophil percentage (Neu%), Mean ± SD	74.86 ± 11.84	73.13 ± 13.93	76.37 ± 9.62	*t* = −1.12	0.267
Albumin (ALB, g/L), Mean ± SD	33.80 ± 6.61	34.17 ± 8.08	33.51 ± 5.20	*t* = 0.39	0.696
Erythrocyte Sedimentation Rate (ESR, mm/h), Median (Q1, Q3)	66.50 (45.50, 74.75)	65.00 (29.25, 70.50)	70.50 (52.25, 76.00)	Z = −1.09	0.278
Complement C3 (mg/dL), Median (Q1, Q3)	82.75 (68.20, 94.53)	83.35 (70.45, 93.15)	82.50 (58.18, 99.72)	*Z* = −0.18	0.857
Rheumatoid Factor (RF, IU/mL), Median (Q1, Q3)	78.00 (35.50, 250.00)	74.50 (35.25, 170.00)	78.00 (39.00, 326.00)	*Z* = −0.25	0.806
Activated Partial Thromboplastin Time (APTT, sec), Median (Q1, Q3)	32.40 (30.20, 35.80)	33.10 (30.20, 35.80)	31.90 (30.20, 35.60)	*Z* = −0.30	0.761
White Blood Cell count (WBC, 10^9/L), Median (Q1, Q3)	8.24 (6.12, 11.22)	7.61 (5.78, 11.57)	8.73 (6.26, 11.05)	*Z* = −0.61	0.541
Platelet count (PLT, 10^9/L), Median (Q1, Q3)	240.00 (193.00, 358.00)	236.00 (165.00, 378.00)	244.00 (200.75, 332.25)	*Z* = −0.29	0.769
High Sensitivity C-reactive Protein (hs-CRP, mg/L), Median (Q1, Q3)	17.42 (2.77, 85.20)	9.44 (1.68, 69.41)	33.14 (6.76, 86.55)	*Z* = −1.29	0.197
Alanine Aminotransferase (ALT, U/L), Median (Q1, Q3)	14.00 (10.00, 25.25)	13.50 (9.00, 21.75)	17.50 (10.00, 27.75)	*Z* = −1.09	0.274
Aspartate Aminotransferase (AST, U/L), Median (Q1, Q3)	19.00 (15.00, 26.00)	19.00 (15.00, 24.50)	19.00 (15.25, 26.00)	*Z* = −0.30	0.767
Triglycerides (TG), Median (Q1, Q3)	1.18 (0.94, 1.93)	1.23 (0.93, 2.16)	1.12 (1.04, 1.72)	*Z* = −0.62	0.535
Total Cholesterol (CHO), Median (Q1, Q3)	4.14 (3.40, 4.79)	4.16 (3.46, 4.70)	4.09 (3.07, 5.15)	*Z* = −0.12	0.907
Serum Creatinine, Median (Q1, Q3)	129.00 (64.00, 383.00)	129.00 (68.00, 318.50)	127.00 (63.00, 391.25)	*Z* = −0.32	0.749
Sex, *n* (%)				*χ*^2^ = 0.12	0.727
Male	35 (49.30)	17 (51.52)	18 (47.37)		
Female	36 (50.70)	16 (48.48)	20 (52.63)		
Smoking status, *n* (%)				*χ*^2^ = 0.18	0.671
Never	47 (66.20)	21 (63.64)	26 (68.42)		
Ever	24 (33.80)	12 (36.36)	12 (31.58)		
Hypertension, *n* (%)				*χ*^2^ = 1.36	0.243
No	50 (70.42)	21 (63.64)	29 (76.32)		
Yes	21 (29.58)	12 (36.36)	9 (23.68)		
Diabetes, *n* (%)				*χ*^2^ = 0.00	1
No	65 (91.55)	30 (90.91)	35 (92.11)		
Yes	6 (8.45)	3 (9.09)	3 (7.89)		
Urine Protein (PRO), *n* (%)				*χ*^2^ = 0.20	0.904
1+	21 (29.58)	9 (27.27)	12 (31.58)		
2+/3+	18 (25.35)	9 (27.27)	9 (23.68)		
Negative	32 (45.07)	15 (45.45)	17 (44.74)		
Lung infection within 1 year of diagnosis, *n* (%)				*χ*^2^ = 0.04	0.845
No	40 (56.34)	19 (57.58)	21 (55.26)		
Yes	31 (43.66)	14 (42.42)	17 (44.74)		
Symptom frequency during treatment, *n* (%)				*χ*^2^ = 6.01	0.014
Persistent	32 (45.07)	20 (60.61)	12 (31.58)		
Intermittent	39 (54.93)	13 (39.39)	26 (68.42)		
Limb numbness or paresthesia, *n* (%)				*χ*^2^ = 8.40	0.004
None	56 (78.87)	31 (93.94)	25 (65.79)		
Present	15 (21.13)	2 (6.06)	13 (34.21)		

t: *t*-test, Z: Mann–Whitney test, *χ*^2^: Chi-square test, SD: standard deviation, M: Median, Q_1_: 1st Quartile, Q_3_: 3rd Quartile.

### LASSO regression analysis

**Figure 1:**
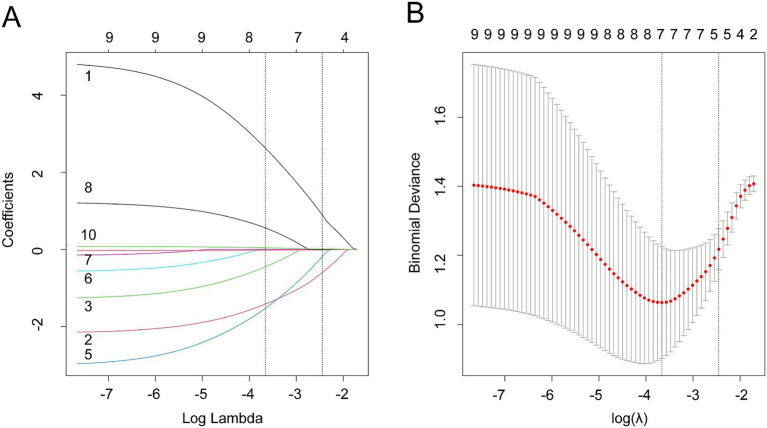
Optimal risk factors identified through LASSO regression analysis. (**A**) LASSO coefficient profile plot for the 10 categorical variables. The coefficient profile is plotted against the logarithmic sequence of *λ* [log(λ)]. (**B**) Selection of the optimal λ parameter using 10-fold cross-validation in the LASSO model. The left dashed line represents the lambda value with the minimum error (lambda.min), while the right dashed line represents the lambda value at one standard error from the minimum (lambda.1-SE).

LASSO regression analysis was conducted to identify the optimal risk factors for pulmonary involvement in AAV patients (see Figure 1). The analysis determined a lambda.min of 0.032, representing the most regularized model within one standard error of the minimum cross-validation error. The coefficient profiles and the selection of lambda are illustrated in Figure 1, providing a clear visualization of the process used to identify the most impactful predictors.

### Multivariable logistic regression analysis

Multivariable logistic regression analysis revealed that intermittent symptom frequency during treatment and the presence of limb numbness or paresthesia were significantly associated with an increased risk of pulmonary involvement (see Table 2). Patients experiencing intermittent symptoms had an over eight-fold increased risk (OR = 8.52, 95% CI: 2.17–33.46, *p* = 0.002), while those with limb numbness or paresthesia exhibited a twelve-fold increase in risk (OR = 12.21, 95% CI: 1.98–75.50, *p* = 0.007). Additionally, age at onset and hemoglobin levels were significant predictors. Each additional year of age increased the risk by 5% (OR = 1.05, 95% CI: 1.01–1.10, *p* = 0.043), and for each 1 g/L decrease in hemoglobin, the risk increased by 3% (OR = 0.97, 95% CI: 0.95–0.99, *p* = 0.038).

**Table 2:** Univariate and multivariate logistic regression analysis of significant factors associated with pulmonary involvement in AAV.

Variables	Univariate logistic regression analysis		Multivariable logistic regression analysis	
	OR (95% CI)	p	OR (95%CI)	p
Symptom frequency during treatment, *n* (%)				
Persistent	Reference		Reference	
Intermittent	3.33 (1.25–8.86)	0.016	8.52 (2.17–33.46)	0.002
Limb numbness or paresthesia, n(%)				
None	Reference		Reference	
Present	8.06 (1.66–39.10)	0.010	12.21 (1.98–75.50)	0.007
Age of onset(years), Mean ± SD	1.06 (1.02–1.10)	0.005	1.05 (1.01–1.10)	0.043
Hemoglobin (HGB,g/L), Mean ± SD	0.97 (0.95–0.99)	0.014	0.97 (0.95–0.99)	0.038

### Nomogram establishment and evaluation of risk prediction model

**Figure 2:**
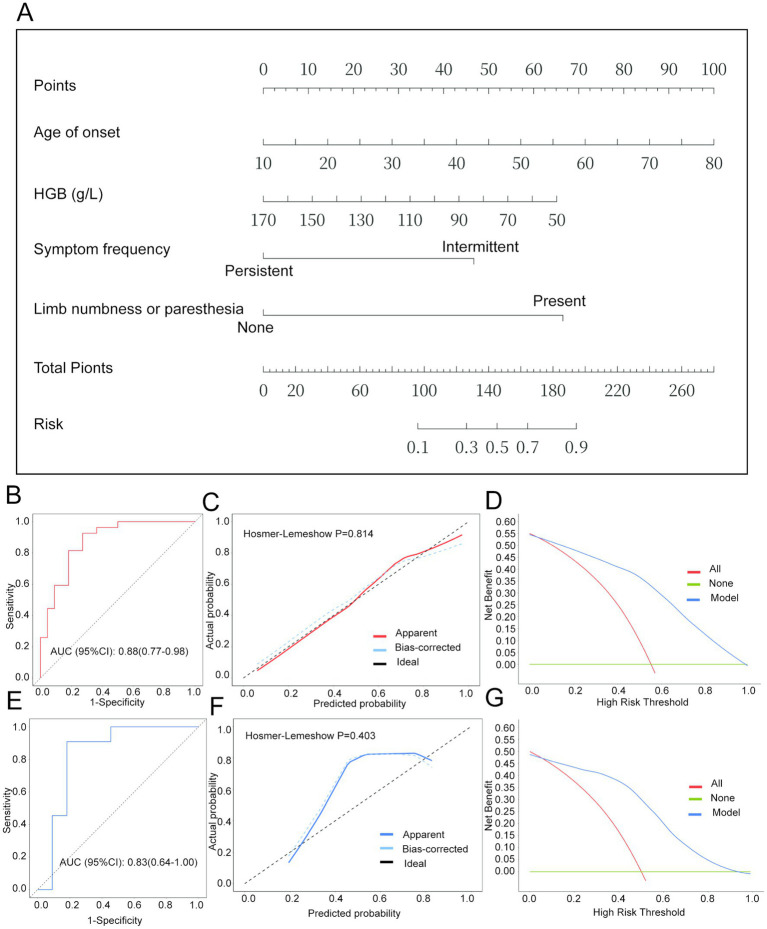
Nomogram and performance evaluation parameters for the risk prediction model of pulmonary involvement in AAV. **(A)** Nomogram for predicting the risk of pulmonary involvement in AAV. To use the nomogram: for each predictor (e.g., Age at onset), locate the patient’s value on the corresponding axis, draw a vertical line upward to the “Points” line to read the assigned points. Sum the points for all predictors to obtain the “Total Points.” Finally, locate the total points on the bottom axis and draw a vertical line downward to read the predicted probability of pulmonary involvement on the “Risk” axis. **(B)** ROC curve for the training set in the risk prediction model. **(C)** Calibration curve for the training set in the risk prediction model. **(D)** Decision curve analysis (DCA) for the training set in the risk prediction model. **(E)** ROC curve for the validation set in testing the risk prediction model. **(F)** Calibration curve for the validation set in testing the risk prediction model. **(G)** DCA curve for the validation set in testing the risk prediction model.

The performance of the predictive model was evaluated using ROC curves, calibration plots, and decision curve analysis (see Figure 2). The nomogram (Figure 2A) visually represents the contributions of key predictors, such as age at onset, hemoglobin levels, symptom frequency, and limb numbness or paresthesia, allowing for individualized risk estimation of pulmonary involvement in AAV patients. The model demonstrated strong discriminative power, with an AUC of 0.88 (95% CI: 0.77–0.98) in the training dataset (Figure 2B) and 0.83 (95% CI: 0.64–1.00) in the validation dataset (Figure 2E). Calibration curves for both datasets (Figures 2C,F) showed excellent agreement between predicted and observed outcomes, with Hosmer-Lemeshow *p*-values of 0.814 (training) and 0.403 (validation), indicating good model calibration. Decision curve analysis (Figures 2D,G) demonstrated a high net benefit across a range of risk thresholds, supporting the model’ s clinical utility in identifying high-risk patients for tailored interventions.

### Restricted cubic spline analysis for age and hemoglobin levels

**Figure 3:**
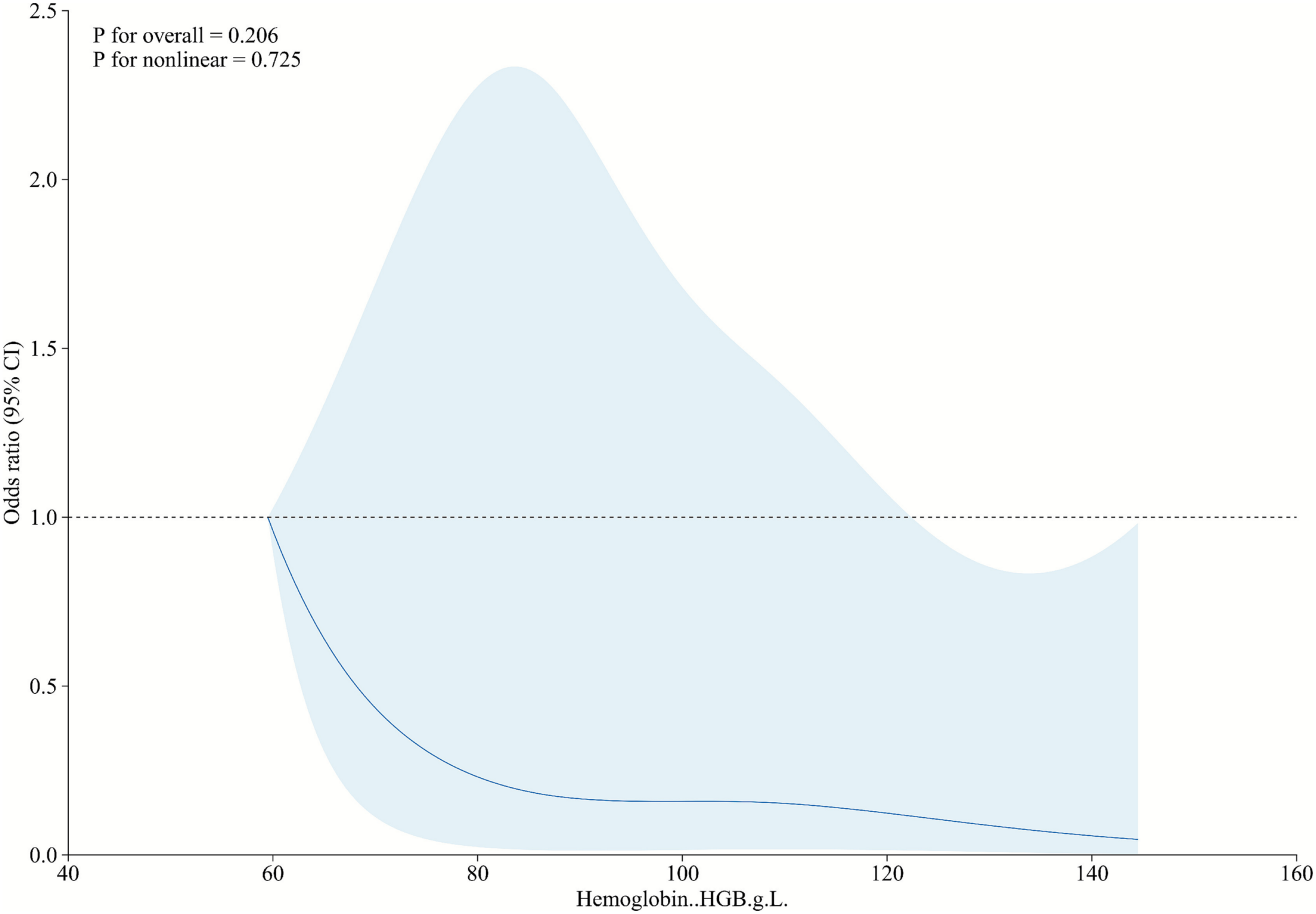
RCS analysis of the relationship between hemoglobin levels and pulmonary involvement outcomes in AAV.

The relationship between hemoglobin levels and pulmonary involvement outcomes in AAV patients was further investigated using restricted cubic spline (RCS) analysis. The RCS plot, presented in Figure 3, demonstrates a non-linear association, with lower hemoglobin levels corresponding to an increased risk of pulmonary involvement. Notably, hemoglobin levels below 100 g/L were significantly associated with higher risk, and a steep escalation in risk was observed at levels under 90 g/L. This analysis, adjusted for age as a covariate, highlights the critical threshold of hemoglobin levels in influencing pulmonary outcomes in AAV patients.

## Discussion

Pulmonary involvement in patients with AAV considerably increases mortality risk, making early identification of high-risk individuals critical for improved prognosis (16, 17). In this study, regression analysis identified age at onset, hemoglobin level, intermittent symptom pattern, and limb numbness or paresthesia as independent predictors of pulmonary involvement. A clinical risk nomogram was created based on these variables. In both training and validation cohorts, the model showed strong calibration and outstanding discriminatory performance (AUC 0.88 vs. 0.83), and decision curve analysis verified its clinical utility over a broad threshold range. By including commonly available clinical signs, this useful tool may help doctors identify high-risk patients early on, allowing for more proactive intervention or closer monitoring and ultimately improving outcomes in this severe subgroup.

In our group, limb numbness or paresthesia was found to be a significant independent predictor of pulmonary involvement (OR = 12.21, 95% CI: 1.98–75.50). The interpretation of this connection necessitates careful evaluation of our study context, even if it has received less attention in previous literature that specifically focused on AAV-related pulmonary involvement. Due to the very small sample size and the modest number of episodes for this particular symptom, the large confidence range indicates significant uncertainty. However, the association’s strength calls for a mechanistic analysis. It could be explained by similar pathways of systemic microvascular injury, where endothelial damage in the pulmonary vasculature occurs parallel to brain ischemia from peripheral or central nervous system vasculitic involvement (18). These neurological symptoms frequently indicate a more severe and widespread kind of vasculitis, which may exacerbate pulmonary damage by increasing immunological activation and systemic inflammation (19). Therefore, this data implies that a straightforward bedside evaluation of neurological symptoms could be a therapeutically effective trigger to raise suspicion for concurrent pulmonary risk, even with the noted statistical uncertainty. This discovery merits validation in larger, prospective cohorts.

Additionally, there was a significant correlation between an intermittent symptom pattern and a higher risk of pulmonary involvement (OR = 8.52, 95% CI: 2.17–33.46). This pattern, which is marked by periods of remission mixed with recurrent episodes of symptoms, may indicate a more erratic or relapsing form of disease activity. Such instability may expose the pulmonary vasculature to recurrent inflammatory assaults, resulting in cumulative endothelial damage and compromised repair mechanisms, which may eventually increase the risk of overt pulmonary involvement. This idea is corroborated by imaging studies, where greater relapse rates in AAV have been independently linked to specific active CT patterns (such as ground-glass opacities) that indicate persistent inflammation (20). A prolonged pattern of symptoms, on the other hand, may be indicative of a more indolent, chronic inflammatory state that develops gradually and may be linked to various pathophysiological adaptations, which could account for the reduced observed risk of acute pulmonary problems. The idea of disease fluctuation as a marker of severity is consistent with observations in other autoimmune conditions, where relapsing–remitting courses are frequently associated with worse long-term outcomes and greater organ damage accrual, even though it has not been thoroughly investigated in the context of AAV-related pulmonary involvement. Recent findings that acute exacerbation is a substantial risk factor for mortality in patients with AAV-associated interstitial lung disease further support this connection (21). Because they can be a subset with increased systemic vasculitic activity and a higher risk of pulmonary involvement, patients with changing symptoms must be constantly monitored.

A lower hemoglobin level was found to be independently linked to a higher probability of pulmonary involvement in our multivariable analysis. There are probably two mechanisms at work in this connection. Lower hemoglobin level often reflects greater disease burden and systemic inflammation in AAV. More importantly, oxygen supply is directly hampered by its lowering (22). Through mechanisms such hypoxia-inducible factor (HIF) activation, the ensuing tissue hypoxia can increase pro-inflammatory cytokines and worsen oxidative stress, creating a vicious cycle that encourages vascular remodeling and parenchymal damage (23, 24). A crucial clinical threshold was highlighted by our data, which showed a sharp increase in risk at hemoglobin levels below 100 g/L, especially under 90 g/L. This implies that aggressively treating anemia might go beyond supportive treatment and possibly break this harmful pathological cycle.

Our investigation indicated that older age at onset was a major risk factor, which is consistent with earlier results (25). There are a number of interconnected reasons why older patients are more vulnerable. First, a significant influence is probably played by immunosenescence, which is defined by a dysregulated inflammatory response and compromised immunological homeostasis. Second, systemic inflammation may be exacerbated by a greater load of comorbidities like diabetes and hypertension. Lastly, the threshold for inflammatory damage may be lowered by age-related structural alterations in the lung (26, 27).

The nomogram developed in this study integrates these readily available clinical indicators to estimate an individual patient’s risk of pulmonary involvement at diagnosis. It is important to emphasize that the probability generated by the model serves as a decision-support tool to inform initial assessment strategies, not as a definitive diagnostic cutoff. The continuous quantitative risk estimate it provides aims to complement, not replace, comprehensive clinical judgment. Based on the risk distribution in our cohort, a predicted probability exceeding 70% may help identify a high-risk subgroup in whom more extensive pulmonary evaluation—such as prompt high-resolution CT imaging and intensified monitoring—could be prioritized. A lower predicted probability may align with a standard assessment pathway. It is critical to note that this suggested threshold is exploratory and should be validated and calibrated in external populations; clinical decision-making must always incorporate the full patient context. Notably, by incorporating indicators such as neurological symptoms, the model enhances its explanatory power and relevance for bedside evaluation. This tool is designed to aid early risk stratification and support individualized management. If further validated in multicenter studies, its integration into electronic health record systems could improve healthcare workflow efficiency; however, its real-world impact must be evaluated through implementation research in actual clinical practice.

This study has several limitations. First, the single-center retrospective design may introduce selection bias, and the modest sample size constrains statistical power and estimate precision, particularly for predictors with wide confidence intervals. However, this challenge is not uncommon in focused studies of ANCA-associated vasculitis, a rare disease with a recently estimated global pooled incidence of 17.2 per million person-years (28). Comparable exploratory prediction model studies in this field have operated with similar cohort sizes (29). To mitigate overfitting within these constraints, we employed rigorous methods including LASSO regularization and bootstrap internal validation. Second, due to the inherent constraints of retrospective data collection, several important variables—including specific ANCA subtypes (MPO- vs. PR3-ANCA) and genetic markers—were not routinely available in our clinical records and therefore could not be analyzed. This may affect the model’s performance across different patient subtypes. Third, the cross-sectional design precludes assessment of causality or long-term predictive ability. Finally, while internally validated, the model requires external validation in prospective, multi-center cohorts to confirm its generalizability and clinical utility.

## Conclusion

In this study, we identified key risk factors for pulmonary involvement in AAV, including intermittent symptoms, limb numbness or paresthesia, age at onset, and hemoglobin levels, and constructed a predictive model based on these factors. The model demonstrated good discriminatory ability, offering a potential tool for integration into clinical workflows to aid in early risk identification and guide personalized treatment. While future multi-center studies are required to validate its applicability across diverse populations, these findings offer useful insights for optimizing management strategies in patients with AAV.

## Data Availability

The raw data supporting the conclusions of this article will be made available by the authors, without undue reservation.
